# Reporter Systems for Assessments of Extracellular Vesicle Transfer

**DOI:** 10.3389/fcvm.2022.922420

**Published:** 2022-06-01

**Authors:** Chaoshan Han, Gangjian Qin

**Affiliations:** ^1^Department of Biomedical Engineering, School of Medicine, University of Alabama at Birmingham, Birmingham, AL, United States; ^2^Department of Biomedical Engineering, School of Engineering, University of Alabama at Birmingham, Birmingham, AL, United States

**Keywords:** exosomes, cell–cell communication, reporter, EVs visualization, recipient cells, extracellular vesicle

## Abstract

Extracellular vesicles (EVs) are lipid bilayer particles naturally released from most if not all cell types to mediate inter-cellular exchange of bioactive molecules. Mounting evidence suggest their important role in diverse pathophysiological processes in the development, growth, homeostasis, and disease. Thus, sensitive and reliable assessments of functional EV cargo transfer from donor to acceptor cells are extremely important. Here, we summarize the methods EV are labeled and their functional transfer in acceptor cells are evaluated by various reporter systems.

## Introduction

Extracellular vesicles (EVs) are lipid-bilayer membrane-enclosed structures released by most if not all cell types in physiological and pathological environments. They are classified into subtypes, including exosomes, ectosomes, and apoptotic bodies, based on the origin of biogenesis, and have different yet overlapping sizes and compositions (1–6). Exosomes (50–150 nm) are formed as intraluminal vesicles of endosomes and released into extracellular space upon endosome and cell membrane fusion, whereas ectosomes (0.1–1 μm) bud out directly from the plasma membrane (6, 7). Apoptotic bodies (50–5000 nm), on the other hand, dislodge from dying and disintegrating cells (8, 9). Loaded with a large spectrum of bioactive agents (i.e., proteins, RNAs, lipids, and metabolites) from donor cells (3, 4), EVs mediate effective transfer of cargos locally to neighboring cells or remotely *via* blood circulation to cells of other organs, thereby impacting the functional states of acceptor cells, tissue homeostasis, and disease pathophysiology (4, 10–16). Upon interacting with acceptor cells, EVs can affect their function *via* triggering membrane-mediated intracellular signaling, fusing with cell membrane to release bioactive cargos into the cytoplasm, or being endocytosed into endosomal system to evade lysosomal degradation through largely unknown mechanisms to regulate cellular activities. For example, exosome-derived ectopic mRNA or miRNA can translate into functional proteins or suppress mRNA translation, respectively, in acceptor cells. Given the often low abundance of exosome-delivered molecules in acceptor cells relative to endogenously expressed biomolecules and the complex intracellular feedback networks, accurate measurements of EV-mediated effects is vital to EV research. Here we summarize a number of reported methods EVs are labeled and their functional transfer are evaluated.

## Extracellular Vesicle Labeling

### Labeling of Isolated Extracellular Vesicles With Chemical Dyes

Labeling of EVs with detectable molecules is widely used for tracking EV biodistribution and uptake into target cells. Various components of EV membrane, particularly lipids and proteins, can be labeled with chemical dyes. Available lipophilic fluorescent dyes include PKH26 (excitation/emission wavelength maxima, λex/λem = 551/567 nm), PKH67 (λex/λem = 490/502 nm), DiO (λex/λem = 484/501 nm), DiI (λex/λem = 549/565 nm), DiR (λex/λem = 750/780 nm), and FM 4–64 (λex/λem = 558/734 nm). While these dyes insert into the membrane lipid bilayer of EVs, generating stable and long-lasting fluorescence signal (17–23), it was reported that at least PKH dyes can trigger EV enlargement as the result of membrane fusion or intercalation (24). Protein fluorescent membrane dyes include carboxyfluorescein succinimidyl diacetate ester fluorescent (25, 26) and maleimide flours (27), which may overcome certain limitations associated with other lipophilic (DiI, PKH67), non-lipophilic (ExoGlow-Vivo), and RNA (SYTO) dyes (27–29).

### Labeling of Isolated Extracellular Vesicles With Aptamers

An aptamer is a short single-stranded DNA or RNA molecule with unique structural features that ensure binding to specific molecular target with known or unknown identity (30). Aptamers are usually selected from synthetic libraries using systematic evolution of ligands by exponential enrichment (SELEX). LZH8, an aptamer selected by using whole HepG2 hepatocytes (31), demonstrates impressive binding affinity to HepG2 cell-derived exosomes (32). Wan et al. linked LZH8 to a “trigger” sequence, so that the LZH8-trigger was able to bind to HepG2-exosomes meanwhile extending by base pairing between the “trigger” sequence and fluorescein (FITC)-conjugated oligos to amplify the FITC signal and enlarge the overall structure, allowing direct flow cytometry analysis of these modified exosomes (32). Nevertheless, whether LZH8 aptamer conjugation affects exosome tropisms or uptake by target cells remain to be elucidated.

### Labeling of Isolated Extracellular Vesicles With Radioisotope or Magnetic Resonance Imaging Contrast Agents

Recently, evidence suggests that radionuclides or magnetic resonance imaging (MRI) contrast fluid can be loaded into the isolated EVs, enabling imaging of administered EVs *in vivo* by nuclear and MRI approaches (33–36). This method is particularly beneficial for deep tissue imaging with the potential of clinical application.

### Tagging of Extracellular Vesicle Surface Protein *via* Genetic Engineering

Exosome membrane contains abundant tetraspanins (CD63, CD81, CD9) and lactadherin, which are often used as exosome biomarkers (37). These molecules were genetically engineered to fuse with fluorescent proteins/bioluminescence-generating enzymes, so the exosomes can be visualized under fluorescent microscope or by supplementation with bioluminogenic substrates (38–41). For example, CD63 fused with pHluorin, a pH-sensitive form of green fluorescent protein (GFP), permits tracking of endogenous EVs in the transparent zebrafish with high spatiotemporal accuracy, leading to the finding that the yolk syncytial layer-derived exosomes are endocytosed by macrophages and endothelial cells of the caudal vein plexus (CVP) in a scavenger receptor- and dynamin-dependent manner, thereby providing the trophic support for CVP growth (42).

Among different bioluminescence-generating enzymes, it appears that NanoLuc or ThermoLuc, when tethered to CD63, are preferred for sensitive imaging and tracking of EVs *in vivo* and *in vitro* (43). Luo et al. generated a transgenic mouse line that expresses CD63-NanoLuc fusion protein specifically in cardiomyocytes, thus cardiomyocyte-derived EVs, and found NanoLuc signals in thymus, testis, lung, and kidney, supporting the notion that cardiomyocyte-derived EVs mediate molecular exchange between heart and other organs (44). Besides CD63, lactadherin was also engineered, such as by fusion with Gaussia luciferase to visualize and track exosomes *in vivo* (45, 46). In a sophisticated system, EV surface was tagged with a membrane-bound biotin acceptor peptide linked outward to *Gaussia* luciferase fusion protein and biotin ligase, thus the EVs can be labeled with supplemented biotin and visualized *in vivo* with duo modal imaging, bioluminescence (with luciferin) and fluorescence (with streptavidin-fluorophore) (47). Lastly, genetic engineering can also achieve radiolabeling. Takakura et al. engineered lactadherin–streptavidin fusion protein on exosomes and used iodine-125 (^125^I)-labeled biotin to label exosomes (48, 49).

Genetic engineering of donor cells to molecularly tag EV surface proteins avoids additional labeling processes and has the advantage of achieving cell-specific EV labeling *in vivo*. Also, it is likely that the engineered proteins, displaying on EV membrane, have significantly lesser untoward effects on the biochemical property of EVs, compared to chemical dyes. However, it should be aware that EVs are diverse in the expression of biomarkers, therefore these methods may be limited by labeling only a certain subgroup of EVs. In addition, although reports were mostly focused on EV membrane proteins, intraluminal soluble proteins, such as exosome-enriched ALIX and TSG101, may also have the potential for tagging, which would indicate the uptake and intracellular trafficking of exosome-derived non-membranous proteins. Lastly, the results obtained from these experiments may still need to be interpreted with care, as the signals are subject to the processing of individual fusion proteins and may not indicate the function of the entire EV proteome.

### Bio-Conjugation of Extracellular Vesicle Proteins

Azide–alkyne cycloaddition (click chemistry) is a powerful tool that permits covalent conjugation, thus tagging, of exosomes. Azide or alkyne group can be incorporated into EVs by supplementing azide or alkyne bearing amino acids (e.g., AHA) (50, 51) or glycans/proteoglycans (52) to EV-producing cells, *in vitro* or *in vivo*, or by directly adding azide or alkyne bearing chemicals to the isolated EVs (53). The click reaction is catalyzed by Cu, but also can occur in Cu-free physiological fluids permitting *in vivo* labeling (54, 55). Diverse imaging modalities (fluorescence, luminescence, radioactive imaging, and MRI) can be adapted to the system for *in vivo* tracking of the labeled EVs. Interestingly, click reactions seem not to affect the size of the exosomes, nor exosome adhesion or internalization in target cells (53).

Notably, David Tirrell group has identified a methionyl-tRNA synthetase L274G mutant (MetRS*), which utilizes the non-canonical azide-bearing amino acid azidonorleucine (ANL) as surrogate of methionine to incorporate into newly synthesized proteins (56). Engineered expression of MetRS* in the neuron enabled ANL-labeling and click-reaction based identification of neuronal specific proteomes *in vivo* (57). Our group introduced MetRS* into mesenchymal stem cells (MSCs) and administered these cells into the ischemic heart of mice supplemented with ANL, followed by serial isolation of azide-labeled (i.e., MSC-derived) proteins from total cardiac protein lysates. MSCs are believed to exert beneficial effects *via* paracrine mechanisms, and our study for the first time revealed MSC proteome real-time *in situ* in the injured cardiac tissue, revealing new insights into MSC mediated cardiac protection and repair (58). We also isolated EVs from ANL-treated MSCs and administered these EVs to mice with surgically induced myocardial infarction. The ANL-labeled (i.e., MSC exosome-derived) proteins were isolated with click-catalyzed alkyne-agarose capture from various organs at different time points and identified with mass spectrometry; the MSC exosomal proteins were also localized histologically *via* fluorescent non-canonical amino-acid tagging (FUNCAT) *in situ*. We found that MSC exosomal proteins distributed in different organs are highly diverse, that ischemic injury significantly augments the tissue intake of exosomes, and that in the injured tissue, the exosomal proteins are predominantly associated with cytosol vs. membrane (51).

Collectively, labeling of EVs has considerably facilitated assessments of their biodistribution and cellular uptake (Table 1), however, it does not allow discrimination between non-functional uptake (lysosomal degradation) and functional transfer or delivery (protein-mediated signaling, mRNA translation, or miRNA repression of target mRNA).

**TABLE 1:** Labeling and reporter systems for evaluation of EV transfer.

Labeling and reporter systems	EV subtype	*In vivo* detection and biodistribution	Functional transfer	Subcellular resolution
Chemical dyes	All EVs	+	−	+
Aptamers	Specific EVs to aptamer	−	−	+
Radioisotope or MRI dye	All EVs	++	−	−
Genetic labeling	Biomarker positive EVs	+	−	+
Cre-loxP	Cre^+^ EVs	−/+	++	++
miRNA targeting reporters	Certain miRNA^+^ EVs	−/+	++	++
CRISPR/Cas9-gRNA reporters	sgRNA^+^ EVs	−/+	++	++

*−, no applicability; −/+, low applicability; +, medium applicability; ++, high applicability.*

## Reporters of Extracellular Vesicle Transfer in Acceptor Cells

While EVs carry various types of bioactive cargos, their mRNA and miRNA activities in the recipient cells are most frequently used to evaluate EV functional transfer (47, 59). An ideal reporter system entails low basal reporter activity in the absence of EVs but highly induced reporter activity when EVs are present. Frequently used reporters are based on the Cre-loxP system, miRNA recognition size on target mRNA 3′UTR, and CRISPR/Cas9 system (Table 1).

### Cre-loxP

The reporter gene is placed downstream of a floxed stop codon in the expression cassette introduced in recipient cells and activated when the cells are transduced with EVs from Cre-expressing donor cells (60). Ridder et al. observed LacZ reporter activation in neurons after taking up EVs carrying functional Cre messenger RNA from immune cells, establishing a unique EV-mediated immune cells to neuron crosstalk (61, 62). In another study, by comparing three Cre reporter mice, Rosa26-LacZ, Rosa26-EGFP, and Rosa26-EYFP, the authors found that Gr1+CD11b+ myeloid-derived suppressor cells (MDSCs) is a major cell population targeted by tumor-released EVs, and that EV transfer augments the immunosuppressive phenotype of these cells (63).

Zomer et al. optimized this Cre-loxP system where the transfer of Cre-bearing EVs induces a switch of DsRed to eGFP expression in the Cre-reporter cells (60). Interestingly, Cre mRNA but not Cre protein was detected in EVs, suggesting that Cre mRNA transfer was primarily responsible for the DsRed-to-eGFP switch (60). Furthermore, the authors applied this Cre-mediated DsRed to eGFP conversion system *in vivo* and found that the recipient tumor cells taking up EVs from highly malignant tumor cells display an augmented migratory and metastatic activity, suggesting that EVs are able to transfer the malignant property of tumors (64). Interestingly, Sterzenbach et al. engineered the expression of Cre protein fused with a WW tag (WW-Cre), which can be recognized by the evolutionarily conserved late-domain L-domain-containing protein Ndfip1, leading to increased Cre protein ubiquitination, thus packing into exosomes; using this system, the authors demonstrated that administered exosomes *via* nasal route reached brain cells, leading to Cre-mediated recombination in mT/GFP mice (65). This study, however, indicated functional delivery of Cre protein through exosomes.

Taken together, the Cre-loxP based reporters have proven that exosomes can mediate functional transfer of Cre mRNA and protein. It should be noted that Cre-loxP system has been used extensively in lineage tracing studies in the past and the Cre mediated reporter expression is rarely in no-Cre expressing cells. Given that exosomes pack predominantly small RNAs or mRNA fragments (66), it is likely that the Cre-loxP system, without enhancement of Cre loading into exosomes, would underestimate EV transfer.

### miRNA Targeting Reporters

miRNAs are predominantly enriched in EVs (59) and well recognized as important effectors responsible for EV induced biological response (66). miRNA target mRNA at specific sequence primarily located in the 3′UTR, inhibiting mRNA translation and inducing mRNA degradation. Dual-luciferase reporter systems are engineered by placing wild-type 3′UTR or miRNA-targeting-sequence mutated 3′UTR downstream of a firefly luciferase reporter; overexpression of miRNA decreases luciferase expression and activity with wild-type 3′UTR but not with mutated 3′UTR (67). Using this system, Thomou et al. engineered an adenoviral vector containing 3′UTR luciferase reporter for human-specific miRNA hsa_miR-302f and transduced mouse liver. They found that expression of pre-hsa_miR-302f but not control miRNA in the brown adipose tissue (BAT) led to >95% reduction of luciferase activity in the liver, indicating EV transfer of hsa_miR-302f from BAT to liver (68). In the mice with adipose-specific knockout of the miRNA-processing enzyme Dicer (ADicerKO), which has a markedly reduced mature miRNAs, the authors found that serum EVs from wild-type mice significantly decreased the level of luciferase activity with a FGF21 mRNA 3′UTR reporter in the ADicerKO liver, further confirming that BAT-derived EVs transfer miRNA to affect gene regulation of the liver (68).

### CRISPR/Cas9 – gRNA Reporters

Recently, de Jong et al. generated an elegant CRISPR/Cas9 based reporter system (69). It is known that Cas9 nuclease mediates non-homologous end joining (NHEJ) repair that creates frameshifts. The reporter construct is driven by CMV promoter; the expression cassette contains genes of mCherry and two alternative reading frames of F2A (a cleavable peptide) fragment and eGFP, which are linked by a “linker-STOP” sequence that contains the specific recognition site of a short single guide RNA (sgRNA) and generates +1 or +2 nt frameshift (eGFP transactivation) at a total frequency of ∼80%. The recipient cells were transduced permanently with the reporter construct, and EV transfer was assessed by co-culture with sgRNA expressing donor cells (close contact or no close contact) or by directly adding donor-derived EVs. The EV-mediated sgRNA transfer permanently activates the expression of eGFP (i.e., mCherry and eGFP double positive). Thus, the system allow visualization of EV-mediated functional sgRNA delivery in single cell level. In a co-culture experiment with cell number ratio of 50:1 (sgRNA donor cells: reporter-transduced recipient cells), an encouraging transfer rate of ∼0.2% was observed (69). Nevertheless, like Cre-loxP based reporter, CRISPR/Cas9 based reporter also causes permanent activation of the reporter gene, the effects are cumulative and not real-time.

Collectively, the reporter systems based on the Cre-loxP system, miRNA and their recognize sites, and the CRISPR/Cas9 system have been used to verify EV mediated intracellular delivery of functional mRNA, protein, and small RNAs in target cells, which are instrumental to our understanding of EV biology.

## Outlook

Conceivably, all direct EV labeling methods (for assessment of EV uptake) and reporter systems (for evaluation of functional transfer) have limitations. Direct EV labeling has the potential of affecting the chemical property of EVs’ membrane, which may alter the EV biodistribution or uptake tropism and dynamics. Genetic engineering of donor cells have the advantage of directly studying endogenous EVs in a tissue specific fashion, however, difficult to distinguish between exosomes, microvesicles, and non-EV associated transport, not to mention the effects of transgenic expression on cellular activities. Current reporter systems, on the other hand, primarily indicate the functional transfer of single molecules (mRNA, protein, or small RNA) in recipient cells, thus subject to the unique dynamics of these individual molecules, and the sensitivity may not be at the same scale as the EV-carried biomolecules to be studied. The choices of reporter systems should be based on the biological questions to be addressed in specific studies and corroborated by other functional readouts. For example, the power of these reporter systems would be significantly enhanced if combined with other cutting-edge technologies, such as single cells sequence or spatial transcriptome analysis. For real-time monitoring of functional mRNA, protein, or sgRNA transfer, some sensitive and specific binary systems, such as the TetR-based transactivators (70) or dCas9-VPR transactivators (71), may be used. The sensitivity of the reporter systems may also be enhanced by increasing the loading of bioactive cargos and/or the membrane fusion, back fusion, or lysosomal escape within recipient cells. Ideally, reporter systems should be able to indicate detailed life-cycle events of EVs, their biogenesis and diversity in donor cells, extracellular navigation, systemic distribution, as well as internalization, intracellular trafficking (between subcellular organelles), cargo delivery or escape, and fate in target cells. The advancement in EV reporter systems will likely provide more powerful and invaluable tools for the field of research in EV biology and therapeutics.

## Author Contributions

CH: conceptual contributions and writing the draft. GQ: conceptual contributions and editing. Both authors contributed to the article and approved the submitted version.

## Conflict of Interest

The authors declare that the research was conducted in the absence of any commercial or financial relationships that could be construed as a potential conflict of interest.

## Publisher’s Note

All claims expressed in this article are solely those of the authors and do not necessarily represent those of their affiliated organizations, or those of the publisher, the editors and the reviewers. Any product that may be evaluated in this article, or claim that may be made by its manufacturer, is not guaranteed or endorsed by the publisher.
